# Neoadjuvant pazopanib and molecular analysis of tissue response in renal cell carcinoma

**DOI:** 10.1172/jci.insight.132852

**Published:** 2020-11-19

**Authors:** Christopher G. Wood, James E. Ferguson, Joel S. Parker, Dominic T. Moore, Jennifer G. Whisenant, Susan J. Maygarden, Eric M. Wallen, William Y. Kim, Mathew I. Milowsky, Kathryn E. Beckermann, Nancy B. Davis, Scott M. Haake, Jose A. Karam, Dante S. Bortone, Benjamin G. Vincent, Thomas Powles, W. Kimryn Rathmell

**Affiliations:** 1 Department of Urology, MD Anderson Cancer Center, Houston, Texas, USA.; 2 Department of Urology, University of North Carolina at Chapel Hill, Chapel Hill, North Carolina, USA.; 3 Lineberger Comprehensive Cancer Center, Chapel Hill, North Carolina, USA.; 4 Department of Medicine, Division of Hematology and Oncology, Vanderbilt University Medical Center, Nashville, Tennessee, USA.; 5 Department of Pathology,; 6 Department of Medicine, Division of Oncology, and; 7 Department of Medicine, Division of Hematology, University of North Carolina at Chapel Hill, Chapel Hill, North Carolina, USA.; 8 Royal Free Hospital, London, United Kingdom.

**Keywords:** Clinical Trials, Oncology, Cancer, Expression profiling, Urology

## Abstract

**BACKGROUND:**

Surgery remains the frontline therapy for patients with localized clear cell renal cell carcinoma (ccRCC); however, 20%–40% recur. Angiogenesis inhibitors have improved survival in metastatic patients and may result in responses in the neoadjuvant setting. The impact of these agents on the tumor genetic heterogeneity or the immune milieu is largely unknown. This phase II study was designed to evaluate safety, response, and effect on tumor tissue of neoadjuvant pazopanib.

**METHODS:**

ccRCC patients with localized disease received pazopanib (800 mg daily; median 8 weeks), followed by nephrectomy. Five tumors were examined for mutations by whole exome sequencing from samples collected before therapy and at nephrectomy. These samples underwent RNA sequencing; 17 samples were available for posttreatment assessment.

**RESULTS:**

Twenty-one patients were enrolled. The overall response rate was 8 of 21 (38%). No patients with progressive disease. At 1-year, response-free survival and overall survival was 83% and 89%, respectively. The most frequent grade 3 toxicity was hypertension (33%, 7 of 21). Sequencing revealed strong concordance between pre- and posttreatment samples within individual tumors, suggesting tumors harbor stable core profiles. However, a reduction in private mutations followed treatment, suggesting a selective process favoring enrichment of driver mutations.

**CONCLUSION:**

Neoadjuvant pazopanib is safe and active in ccRCC. Future genomic analyses may enable the segregation of driver and passenger mutations. Furthermore, tumor infiltrating immune cells persist during therapy, suggesting that pazopanib can be combined with immune checkpoint inhibitors without dampening the immune response.

**FUNDING:**

Support was provided by Novartis and GlaxoSmithKline as part of an investigator-initiated study.

## Abstract 2

Neoadjuvant treatment with pazopanib results in tumor response in clear cell renal cell carcinoma, and lends insight into genetic and immunological effects of these inhibitors.

## Introduction

Renal cell carcinoma (RCC) is the most lethal of urologic malignancies, accounting for an estimated 65,340 new cases and 14,970 deaths in the United States in 2018 (1). Surgical resection for clinically localized disease remains the mainstay for curative intervention. However, approximately 20%–40% of patients will develop disease recurrence, and two-thirds of these patients will have their disease recur within the first year after nephrectomy (2). Although sunitinib was approved in the adjuvant setting in the United States based on recurrence free survival (3), in the neoadjuvant setting, no therapies have been approved (4). Thus, although significant improvements have been made in the management of metastatic disease, risk reduction at the point of surgery is still an active area of interest.

RCC has undergone a renaissance of treatment strategies in the past decade, first with the introduction of targeted therapies that have the potential to downsize tumors, delay disease progression, and markedly improve survival (5–7). In particular, therapies directed toward the VEGFR have consistently demonstrated promise in promoting reductions in tumor burden, thus providing a major mechanism for relief of pain and other tumor-related symptoms. Pazopanib, an oral angiogenesis inhibitor whose targets include VEGFR, the platelet-derived growth factor receptor (PDGFR), and c-Kit (8), received FDA approval for the treatment of patients with advanced RCC based on a significant improvement in progression-free survival (PFS) compared with placebo (9.2 months versus 4.2 months, *P* < 0.001), with an overall response rate (RR) of 30% (5).

A majority (88%) of the patients enrolled on the randomized phase III trial that led to FDA approval of pazopanib had prior nephrectomy, thus limiting the generalizability of those results to the neoadjuvant setting. A recent study in patients with metastatic disease evaluated the safety and efficacy of preoperative pazopanib, and it reported a 13% RR and 84% clinical benefit rate with manageable toxicities (9). Based on the demonstrated success in metastatic RCC, it is plausible that patients with locally advanced disease would also derive benefit from neoadjuvant pazopanib. Moreover, the second major breakthrough in the treatment of RCC has been the introduction of immune checkpoint blockade (ICB). ICB has broad indications in the treatment of metastatic disease on the basis of RR and overall survival data (10). Furthermore, combinations of angiogenesis inhibitors with ICB are undergoing aggressive investigation (11–13). As ICB therapies are making their way into the perioperative setting, with major phase III clinical trials underway (14), it is essential to understand the potential effect of angiogenic inhibitors to the tumor and the tumor microenvironment. Thus, we designed this phase II study to evaluate the safety and efficacy of neoadjuvant pazopanib in patients with clinical stage II or greater localized disease.

As an exploratory objective, we described the association between specific molecular features of RCC and tumor response. Clear cell type RCC (ccRCC) is a disease dominated by only a few high-frequency gene mutation events. Mutations in the von Hippel-Lindau (VHL) tumor suppressor are common, in combination with chromosome 3p loss of heterozygosity (15). Mutations in several other 3p genes occur secondarily in *SETD2*, *PBRM1*, and *BAP1*, all identified via high-throughput sequencing studies (16–19). Genes such as *TP53*, *PTEN*, and *mTOR* are mutated in approximately 5% of cases. The effect of VEGF targeted therapy on the composition of mutational clones within individual tumors is unknown. Theoretically, resistant clones may become more apparent after treatment due to clonal selection. Therefore, we performed temporally separate tumor sequencing analyses in order to examine the effect of treatment on the tumor mutational spectrum as a result of exposure to pazopanib. We also examined pre- and posttreatment tumors using RNA sequencing analysis to observe effects on the general transcript profile and tumor infiltrating immune signatures.

## Results

### Patient characteristics and study schema.

**Figure 1:**
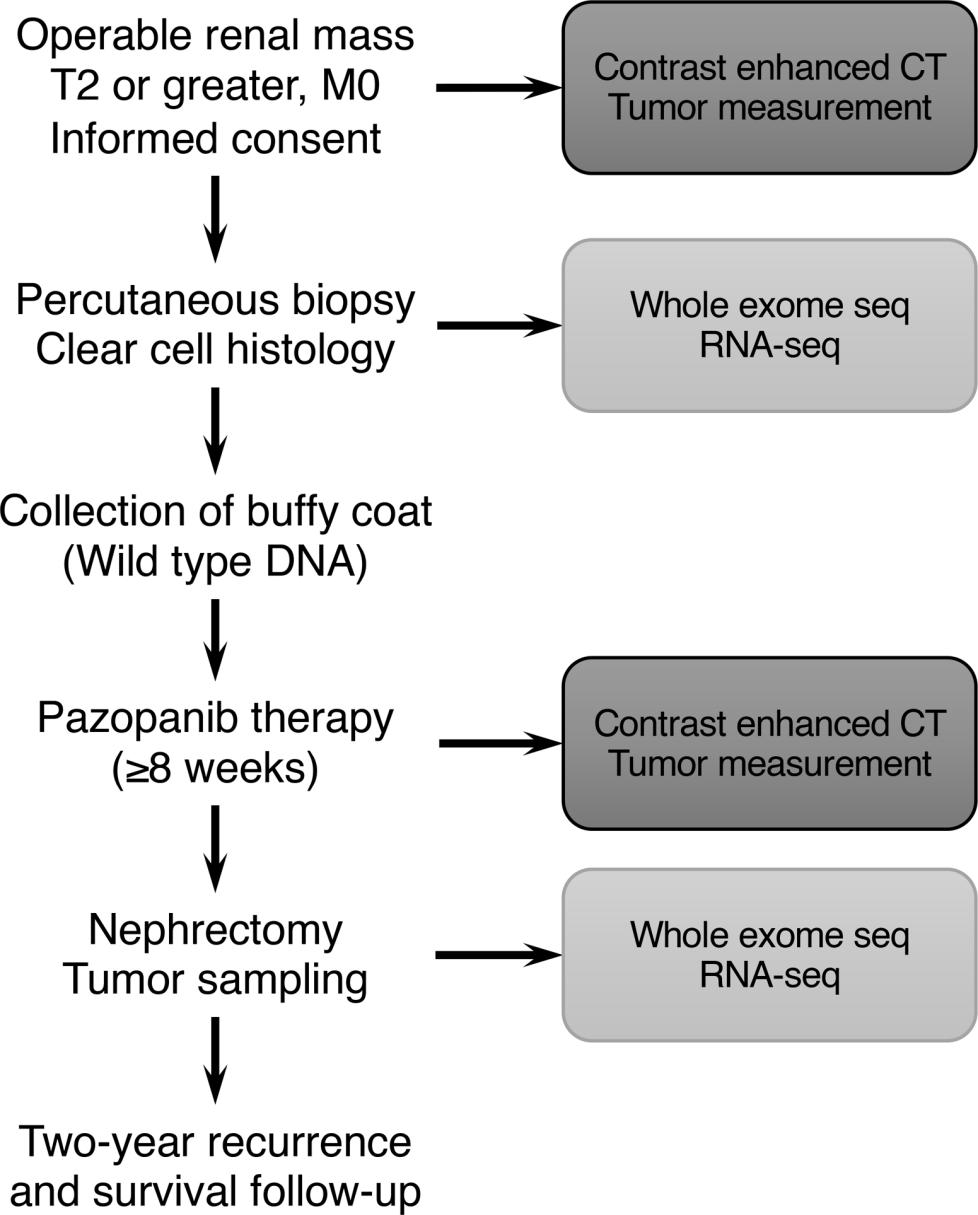
Treatment and molecular biomarker analysis schema. Patients enrolled in this study were identified in the urological oncology clinic with nonmetastatic stage II or greater disease. All patients underwent a percutaneous biopsy to confirm clear cell histology and donated whole blood for genomic DNA comparison. Patients were treated with at least 8 weeks of pazopanib and evaluated by repeat imaging before nephrectomy. DNA was prepared for whole exome sequencing from the whole blood buffy coat, the pretreatment biopsy, and the posttreatment nephrectomy specimens. RNA was prepared for transcript analysis from pretreatment biopsy and nephrectomy specimen.

Between July 2011 and October 2014, 21 patients were consented and enrolled. Baseline demographics and disease characteristics are listed in Table 1. The median age was 61 years (range:, 37–75 years), 86% (18 of 21) were male, and 62% (13 of 21) had hypertension at baseline. Tumors ranged in size from 3.4 cm to 11.7 cm in largest diameter, and a majority (52%) were stage IIIA. Eleven patients received treatment for 10–12 weeks, based on an initial schedule that called for 12 weeks of drug treatment. No significant difference in response was observed for any of the demographic subgroups. The study was amended to allow 8 weeks of treatment, due to challenges meeting target enrollment. One patient received only 6 weeks of treatment due to dose interruption. Figure 1 depicts the study schema.

### Efficacy.

**Figure 2:**
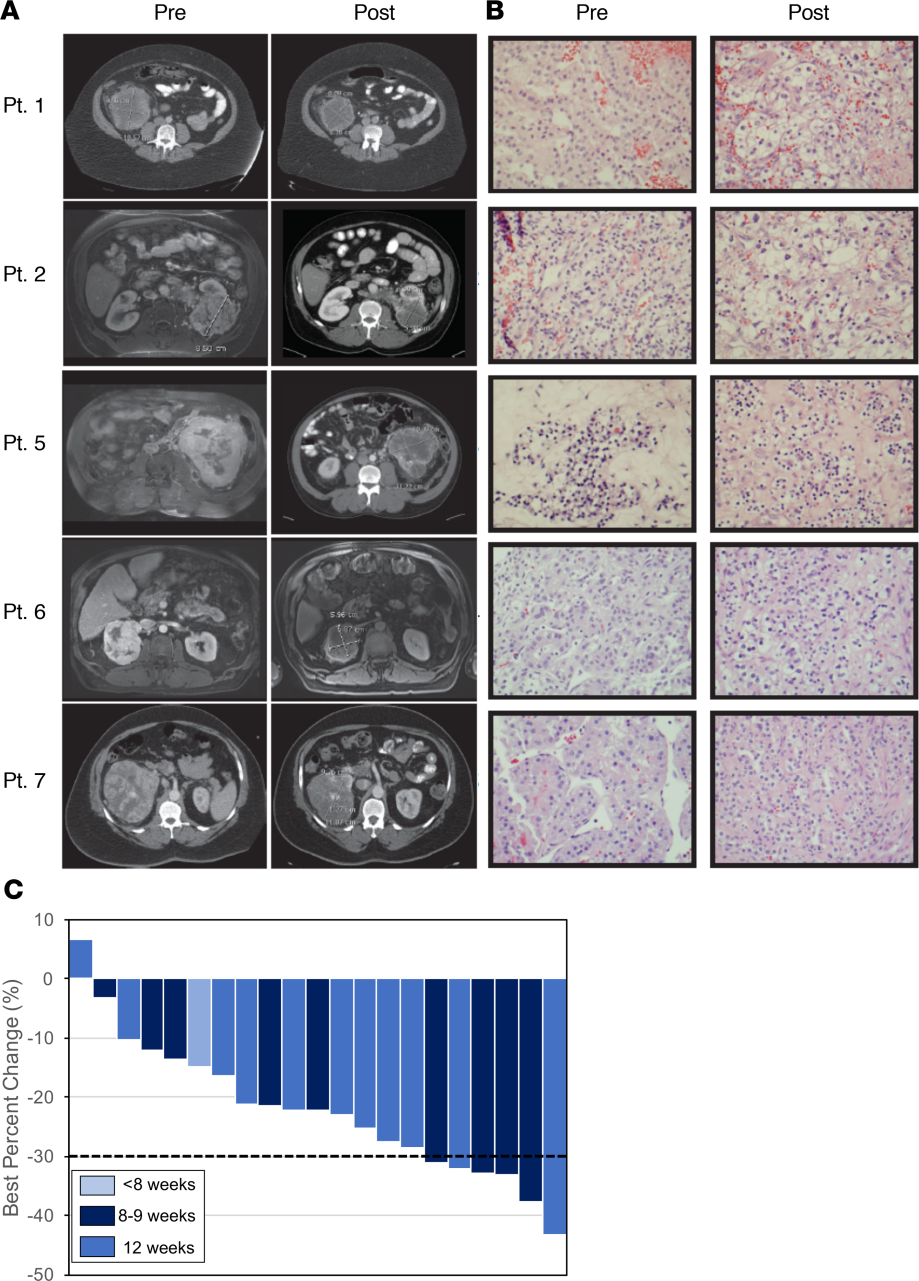
Neoadjuvant treatment with pazopanib resulted in encouraging clinical activity in patients with treatment-naive, locally advanced clear cell RCC. (**A**) Representative examples of pre- and posttreatment CT images of a partial responder (left, patient [Pt.] 5) and a patient with stable disease (left, Pt. 6), respectively. RECIST v1.1 longest dimensions are shown in each panel. Changes in tumor density were not captured in response assessment. (**B**) Histology for each patient is shown. (**C**) Best percent change from baseline is shown for each patient, with the color scheme representing duration of neoadjuvant treatment. Dashed line is the RECIST v1.1–defined PR, showing 6 patients exhibited a PR on study. Additionally, the duration of neoadjuvant treatment has little effect on tumor regression.

Of the 21 patients enrolled, 8 achieved a partial response (PR) for a 38% RR. The other 13 patients achieved stable disease (SD) by Response Evaluation Criteria in Solid Tumors (RECIST). Representative imaging and histology of 5 patients is provided in addition to a graphical representation of best response (Figure 2). The median reduction in tumor size after treatment was –22%; (range, –44% to +7%). No difference in tumor response was observed between patients receiving 8–9 weeks of treatment versus those treated for 10–12 weeks (*P* = 0.82). Representative histological sections before and after treatment are provided to demonstrate that substantial changes in the tumor histology were not observed. At 1 year, RFS was.83 (95% CI, 0.57–0.94) and OS was.89 (95% CI, 0.62–0.97). Treating urologists were asked to comment on planned surgical approach before the start of treatment, and the applied surgical procedure remained unchanged after treatment for all patients. The majority of tumors were removed by radical nephrectomy (19 of 21), and 2 were removed by partial nephrectomy. Six patients underwent laparoscopic nephrectomy; the remainder were completed via an open approach.

### Safety.

Adverse events experienced by at least 3 patients are listed in Table 2. Most common treatment-related toxicities were fatigue (71%), hypertension (57%), dysgeusia (52%), diarrhea (48%), nausea (43%), elevated aspartate aminotransferase (38%), elevated alanine aminotransferase (33%), and anorexia (33%) — the majority being grade ≤ 2. However, 7 patients experienced grade 3 hypertension, 2 patients had grade 3 elevated alanine aminotransferase, and 1 patient had grade 3 elevated aspartate aminotransferase. Other grade 3 events are listed in Table 2. No grade 4 or 5 treatment-related events were reported. No patients withdrew due to toxicity, although dose interruptions occurred for all grade 3 and bothersome grade 2 toxicities. Planned surgery was not delayed due to any pazopanib-related adverse events.

### Molecular analysis of allele frequency.

Tumor specimens were collected before and after treatment using the schema outlined in Figure 1. High-throughput exon sequencing revealed an average of 190 mutations (range, 100–240) in each tumor, which were recorded for each pre- and posttreatment specimen using the whole blood sample sequence as a reference. In order to confirm that significant differences at the level of copy number gains and losses were not observed in the sample pairs due to either sampling effects or therapeutic exposure, we generated copy number features from the sequence read depths (Supplemental Figure 1; supplemental material available online with this article; https://doi.org/10.1172/jci.insight.132852DS1). This highly concordant genome map indicates that the samples did not differ as clones at the level of larger-scale copy number events. Further evidence is provided by the Kolmogorov-Smirnov test of distributions limited to the shared mutations. The lack of significance for distributional differences in these shared mutations lends support that the significant differences seen in all mutations were not substantially affected by tumor cellularity.

**Figure 3:**
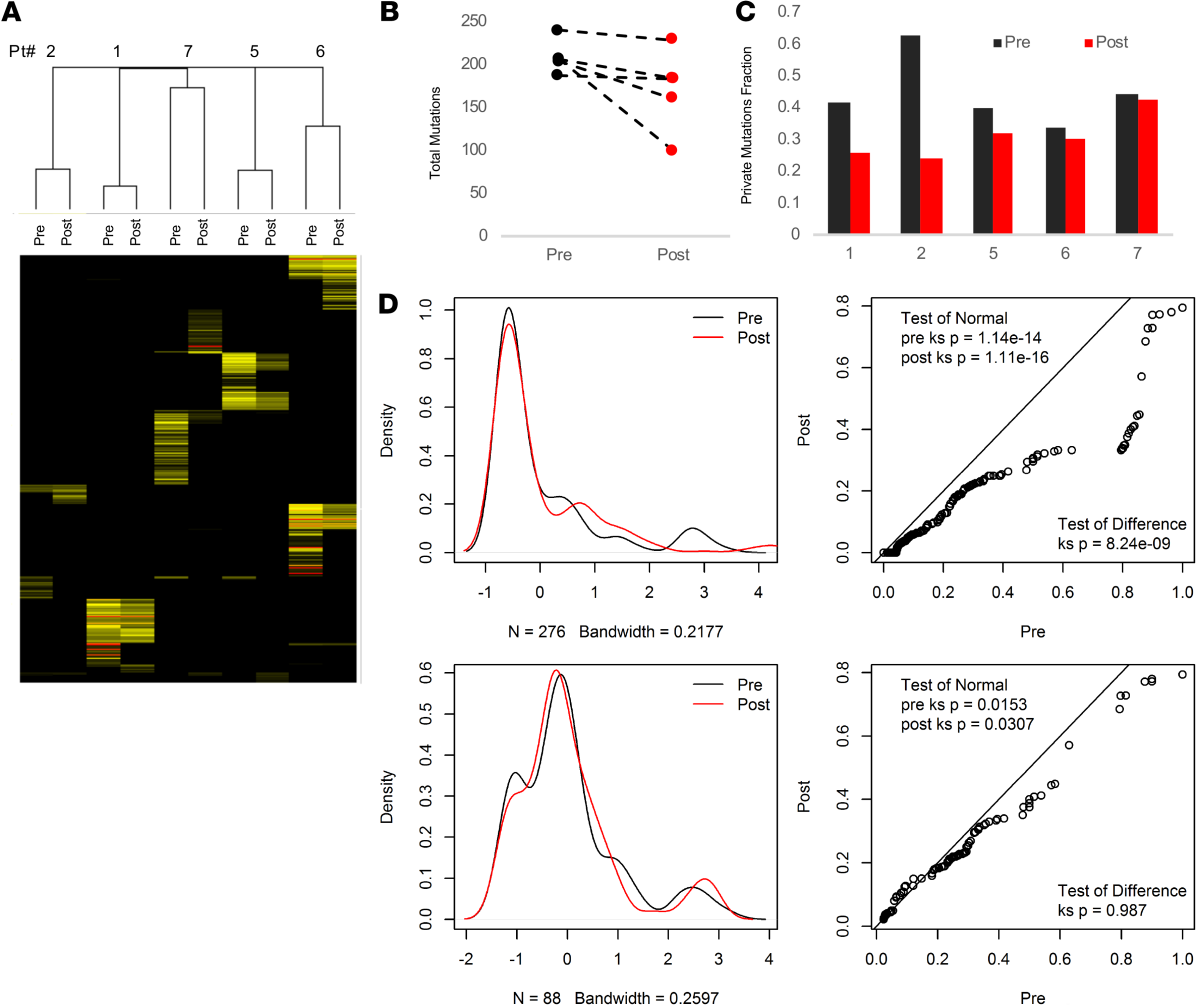
Results of the DNA allelic frequency analysis. (**A**) Hierarchical cluster analysis of mutations from pre- to posttreatment samples reveals close clustering of corresponding pre- and posttreatment samples. Each tumor is annotated along *x* axis. MAF, mutant allele frequency. (**B**) Overall total mutations are decreased in posttreatment tumors. (**C**) The number of private mutations for each sample was divided by the overall number of mutations between the pairs to yield the fraction of private mutations: a reflection of increasing clonality in the post-treatment tumors. (**D**) Density plots comparing all mutations with shared mutations only (private mutations excluded) shows that differences between samples are due to private mutations.

Using an unsupervised clustering analysis, we observed complete pairing between pre- and posttreatment (Figure 3A). This finding suggests that, in spite of substantial differences in the mutations in any given sample, core mutations display a level of consistency within the same tumor, even when the specimens are separated by distance, time, and treatment exposure.

Moreover, although the frequency varied between cases, the fraction of private mutations was reduced in all of the pairings in the posttreatment sample (Figure 3C). Private mutations are defined here as mutations that are uniquely observed in a single tumor sample and not shared by any other tumor sample from the same subject. This may suggest that there is a selective reduction in genetic diversity in samples collected following treatment with pazopanib. The change in total mutation frequency between pre- and posttreatment for each sample is shown in Figure 3B. Density plots of the overall and private mutation profiles for the samples in aggregate demonstrate that the reduced diversity stems from reductions in private mutations, overall preserving the mutations that make up the shared set (Figure 3D). For each pair, we compared the pre- and posttreatment mutant allele frequency using a scatter plot method. It appears that the increases in mutant allele frequencies were largely driven by exclusion of private mutations (Supplemental Figure 2).

### Transcriptome profile changes with neoadjuvant pazopanib therapy.

**Figure 4:**
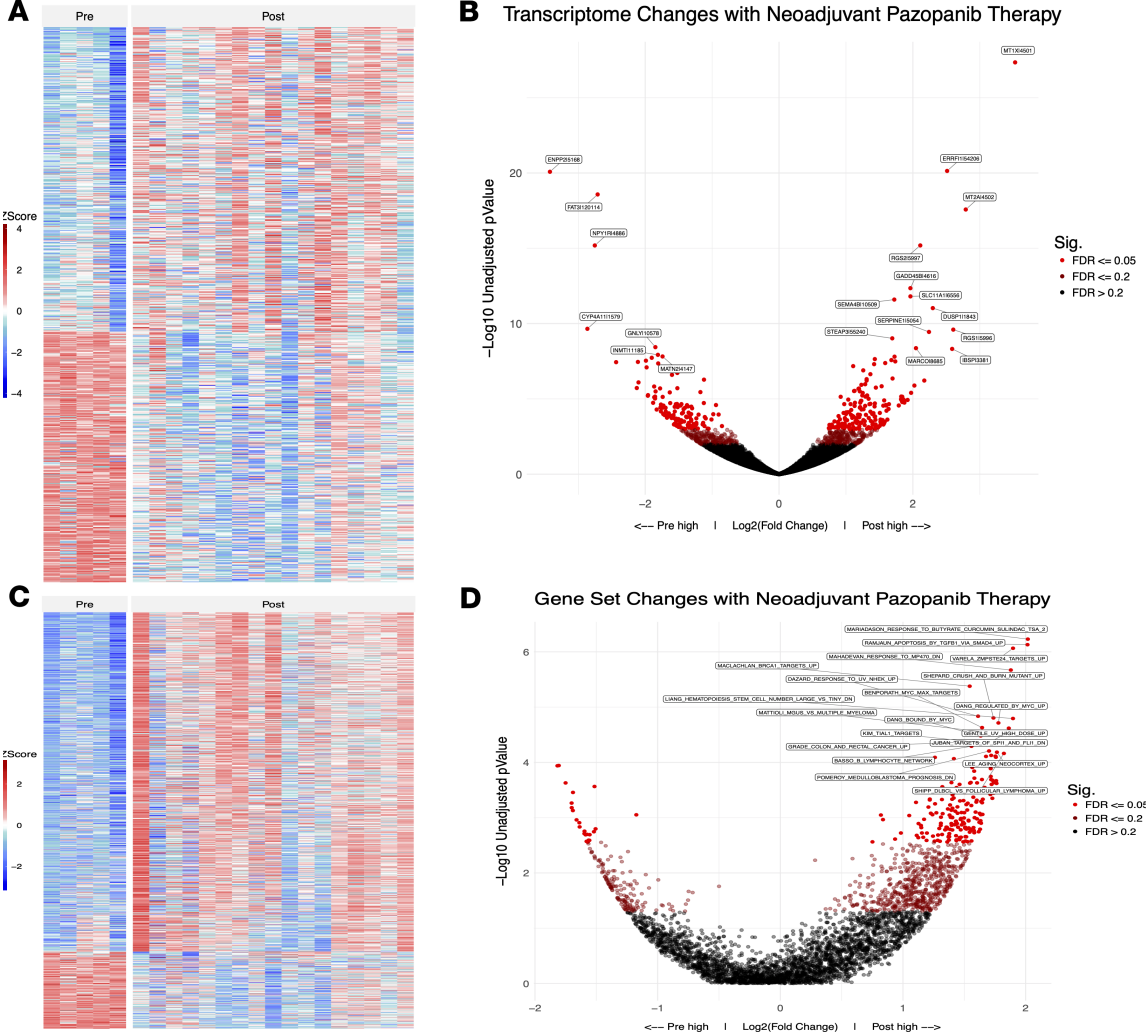
Transcriptional changes with neoadjuvant pazopanib therapy. (**A**) DESeq2 was used to perform differential gene expression analysis on unnormalized gene counts from patients before and after pazopanib treatment. Heatmap shows *z* scores of genes significantly different after Benjamini-Hochberg FDR correction (*P* ≤ 0.2) ordered by fold change. (**B**) Volcano plot shows all DESeq2 gene results, graphing *P* value over change. Color indicates FDR corrected *P* value of gene. The 20 most significant genes are labeled. (**C**) Single-sample gene set enrichment analysis (ssGSEA) was performed on upper-quartile normalized gene expression data from patients in **A**. The primary gene sets used were MSigDB’s chemical and genetic perturbations, KEGG, and oncogenetic signatures. As in **A**, *z* scores of gene sets significantly different (FDR-corrected *P* ≤ 0.2) are shown. (**D**) All ssGSEA gene sets from analysis in **C** are shown with the highest 20 genes sets labeled.

In order to better understand the effect of neoadjuvant pazopanib on the tumor and its microenvironment, we performed RNA sequencing of tumor specimens. A number of genes were significantly upregulated and downregulated by neoadjuvant pazopanib (Figure 4, A and B). Genes with significantly increased expression after therapy included the metallothioneins *MT1X* and *MT2A*, a divalent cation transporter protein (*SLC11A1*); *ERRFI1*, G protein receptor genes that have been implicated in preventing cardiac hypertrophy (*RGS2*, *RGS1*); and *GADD45B*, a gene found to have increased expression with stress-induced growth arrest. Genes significantly downregulated with pazopanib therapy included *ENPP2* (autotaxin), which has been implicated in angiogenesis and tumor cell motility; the cadherin *FAT3*; and the kidney-enriched cytochrome monooxygenase *CYP4A11*. To evaluate whether gene expression patterns were consistent with activation of specific biological processes, we performed gene set enrichment analysis (GSEA) (20). Gene sets with increased expression following neoadjuvant pazopanib included those associated with cellular stress responses (Figure 4, C and D).

Because ccRCC bears such a close association with hypoxia-induced transcript signatures, a specific analysis of signatures linked with hypoxia signaling was undertaken (Supplemental Figure 3). Among common signatures of hypoxia signaling, only a transcript signature of migration and invasion was significantly upregulated in the posttreatment tumor specimens (*P* < 0.005).

### Stability of immunogenomics features with neoadjuvant pazopanib therapy.

**Figure 5:**
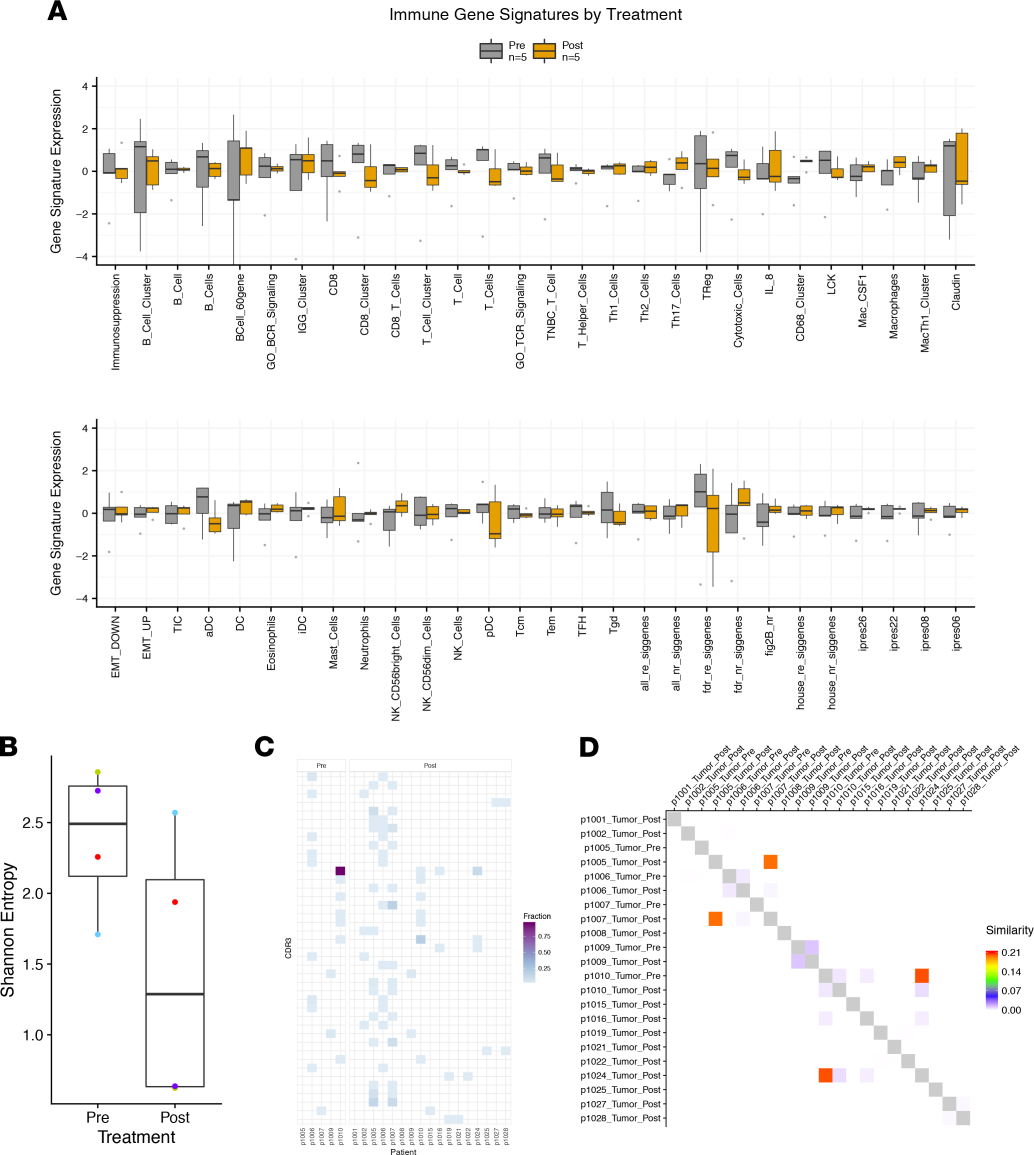
Immune features pre and post neoadjuvant pazopanib therapy. (**A**) Immune gene signatures were made from log_2_ normalized gene counts. Two-tailed *t* test comparisons were made between matched patient pre- and posttreatment samples. No signatures were significant after Benjamini-Hochberg FDR correction. (**B**) Shannon entropy was calculated on TRB CDR3 counts. Points are colored by patient. Pre- and posttreatment results were not significantly different (paired *t* test, *P* = 0.18). (**C**) Public IgH CDR3 are represented on the *y* axis. Heatmap colors indicate the fraction of total counts a CDR3 represents out of the total IgH CDR3 counts for each a sample. (**D**) Morisita-Horn index was calculated on the fractional expression of all IgH CDR3 for each sample combination.

We hypothesized that neoadjuvant pazopanib may elicit changes in the tumor immune microenvironment that would be evaluable via immunogenomics analysis. We did not find any immune gene signatures with significantly different expression comparing pre- and postpazopanib tumor gene expression (Figure 5). T cell receptor (TCR) repertoire diversity measured by the Shannon Entropy index showed some decreased diversity after treatment; however, the difference was not statistically significant due to diversity in a single patient increasing between pre- versus posttreatment (Figure 5B, blue points). There were also substantial differences in dominant and subdominant TCR clonotypes expressed, both within individual patients and between patients (Figure 5C). However, in 2 patient pairs after treatment, there was increased TCR clonotype sharing (i.e., the same clones present in both members of the pair) (Figure 5D).

### Association of transcriptome features with clinical response.

**Figure 6:**
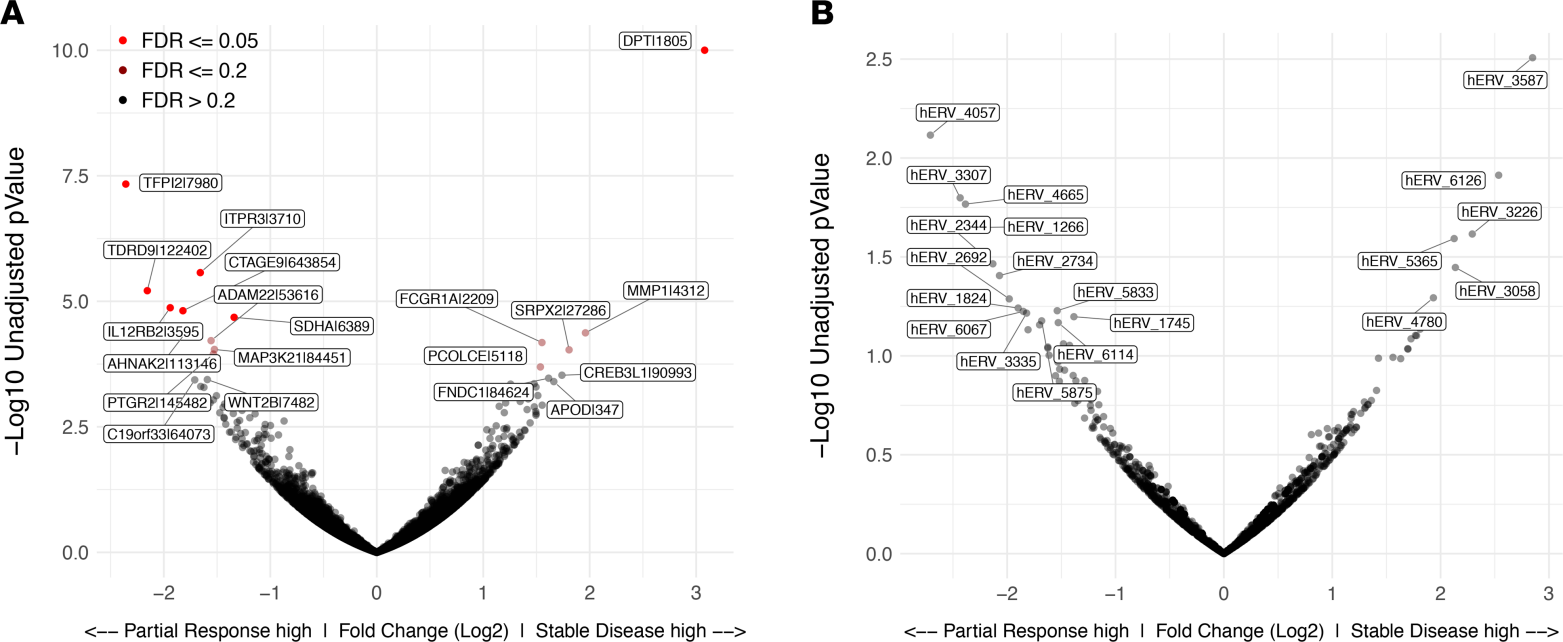
Transcriptional changes with neoadjuvant pazopanib therapy. DESeq2 was used to perform differential gene expression analysis on unnormalized gene counts between pretreatment samples of patients (*n* = 15) showing stable disease and patients (*n* = 6) showing partial response to pazopanib treatment. Volcano plot shows all DESeq2 (R package v1.14.1; ref. 38) gene results, graphing *P* value over fold change. Color indicates FDR-corrected *P* value of gene. The 20 most significant genes are labeled. (**A** and **B**) Typical non-hERV encoding genes (**A**) and hERV genes (**B**).

We conducted an exploratory analysis to determine whether pretreatment gene expression or gene set enrichment was associated with clinical response. Comparing the 2 outcomes in the study across the neoadjuvant time frame (SD or PR), the most significant differentially expressed gene (FDR < 0.2) was the procadherin *PCDHB5*, which had increased expression in patients who achieved PR (Figure 6). Additionally, in light of recent findings linking expression of elements of human endogenous retroviruses to response to checkpoint immunotherapy (21, 22), we examined the transcript data for evidence of any such association, finding none.

## Discussion

The role of treatment with antiangiogenic agents, such as pazopanib, has been well established in patients with metastatic RCC. Therefore, it is plausible that neoadjuvant regimens would elicit similar benefit before nephrectomy and perhaps extend RFS. The aim of this study was to evaluate the safety and efficacy of neoadjuvant pazopanib in patients with localized, untreated ccRCC. Overall, neoadjuvant pazopanib was safe, with most treatment-related toxicities being grade ≤ 2. Frequency and severity of toxicities were similar to those previously reported with pazopanib (5, 9, 23). Fatigue; hypertension; gastrointestinal toxicities, such as dysgeusia, diarrhea, and nausea; and elevated liver enzymes were most common adverse events and were manageable with conservative measures.

Neoadjuvant pazopanib resulted in 8 tumor PRs (38%, 8 of 21). Responses observed in this study are similar to the study from Rini et al. that reported a 36% RR in patients with localized ccRCC who underwent 8–16 weeks of neoadjuvant pazopanib (23). Furthermore, these observed responses were similar to that reported in the phase III randomized study (30% RR by independent review) that led to pazopanib approval for patients with metastatic disease (5). Collectively, these results suggest that neoadjuvant pazopanib is just as effective for the treatment of patients with localized ccRCC.

No significant difference in response was observed, despite patients receiving drug over a time ranging from 8 to 12 weeks, although the optimal duration remains unknown. These findings were consistent with the findings of Rini et al., which reported that most of the substantial responses that changed surgery from radical to partial nephrectomy was observed after 8 weeks (23). However, the limitations of the current and previous neoadjuvant studies highlight the fact that the clinical utility of neoadjuvant pazopanib will need further evaluation in larger, prospective cohorts — ideally, randomized studies that evaluate clinical outcomes of neoadjuvant pazopanib followed by nephrectomy versus nephrectomy alone. Nonetheless, neoadjuvant treatment with pazopanib is effective at reducing tumor size, potentially leading to less complicated surgical approaches in patients with localized ccRCC.

This study, which consisted of molecular analyses, identified strong intratumoral correlations in the mutational spectrum of prepazopanib-treated and postpazopanib-treated specimens. This consistent mutational pattern of exclusion of private mutations and enrichment of the driven genes seen in the paired biopsies has a vanishingly low random probability. Indeed, the 5 cases with paired samples cluster together. This finding does not contradict the heterogeneity studies previously reported (24, 25). While tumor heterogeneity exists in both models, our examination of tumor specimens separated by time, space, and treatment exposure indicates that these factors have the potential to influence the mutation spectrum of a tumor.

Treatment appeared to enrich for relevant mutations commonly associated with RCC. The majority of tumors demonstrated a reduction in overall mutation number and an increase in mutant allele frequency, rather than evidence for adaptations that increase heterogeneity through further mutation or other genomic instability. Whether this adaptation involves a selective elimination of cells bearing passenger mutations, or an enrichment for a clonally derived set of tumor cells, it would appear that the exposure to a relatively short period of anti-VEGFR therapy selects for a unique set of tumor cells in each tumor. It cannot be determined if this reflects sensitivity to therapy of a specific tumor cell subset or a feature of emergent resistance — only that the mutations identified in the posttreatment sample remain relevant and are not indicative of the expansion of a highly heterogeneous tumor cell pool. A third tumor biopsy at clinically apparent resistance would be required to answer this question.

The concept that tumor selection can enrich for important driver mutations is not new in RCC. Recently, Peña-Llopis et al. demonstrated that tumor cells grown as patient-derived xenografts enriched sufficiently to allow the discovery of *BAP1* as a leading driver mutation (17). The process of tumor survival in the setting of VEGF-targeted therapy exerts a different but similar set of external forces, which may also enrich for aggressive clonal subsets. Pazopanib therapy may similarly favor the expansion of clones, possibly owing to the altered extracellular environment or depletion of oxygen and nutrient resources, resulting in the enrichment of clones. This may be explained by the increased mutation allele frequency, through a selective exclusion of a subset of the tumor cells, thus eliminating the noise of highly variable passenger mutations.

We show that neoadjuvant pazopanib led to decreased genetic diversity via a contraction of tumor clonotypes recovered from whole exome sequencing (Figure 3). Additionally, although the T cell content was slightly reduced, we do not have sufficient data to imply that this reflects T cell repertoire restriction in response to treatment. In a recent analysis of genomics data from melanoma patients treated with PD-1 inhibition (nivolumab), genomic contraction on therapy was significantly associated with achievement of partial or complete response (26). In non–small cell lung cancer, tumor genetic heterogeneity was associated with primary resistance to immune checkpoint inhibition (27). Future studies using single cell analysis will be necessary to address this issue directly. Finally, while we do not hypothesize that pazopanib works primarily through an immunological mechanism, should a relationship between genetic homogeneity and response be confirmed, neoadjuvant therapy may be an effective venue for combination treatments of VEGF receptor TKI with immune checkpoint inhibition and should be evaluated further.

Ultimately, this exploratory study provides information on 2 aspects of RCC tumor genome biology. First, samples within a tumor, even temporally separated by a therapeutic intervention, cluster together, revealing that each tumor has a fairly unique profile of mutations that separates it from other tumors with similar histological features. Therefore, biomarker research to define tumors using discrete specimens is not futile (28), despite the presence of tumor heterogeneity. Second, this exploratory analysis revealed that genomic heterogeneity is reduced within samples following treatment with VEGF-targeted therapy, suggesting an alteration in the process of clonal evolution with elimination of specific tumor cell subsets sensitive to treatment. The posttreatment tumor may therefore be more representative of the clones responsible for driving the cancer or more relevant to the disease process. Genetic biomarker analysis after a specific period of targeted therapy should be considered as medical practice moves toward personalized therapy.

This study had several limitations. Given the small sample size, it is difficult to compare the frequency of common driver mutations to larger data sets. In addition, the small size of the biopsy samples, obtained by core biopsy, expose the specimens to spatial heterogeneity. Second, the low frequency of *VHL* mutations in our set may seem surprising in light of the high frequency of these mutations that is seen in some series. However, our methodologies do not capture hypermethylation as a cause of *VHL* inactivation, and in the many large data sets, the combined *VHL* mutation and methylation frequency is just over 50% (29). Nonetheless, we were able to find statistically and clinically significant findings.

In conclusion, our results, albeit in a small sample, suggest that neoadjuvant pazopanib is safe and efficacious to produce tumor reduction in ccRCC patients who are treatment naive and have localized disease. Additionally, exploratory genomic analyses showed that the majority of tumors demonstrated a reduction in overall mutation number and an increase in mutant allele frequency without altering immune expression signatures substantially, providing evidence for further investigation of specific genetic biomarkers of tumor response.

## Methods

### Study design and patient selection

This multiple-institution, single-arm phase II study (NCT01361113) investigated neoadjuvant pazopanib in patients with localized ccRCC. Pazopanib was orally administered in 800 mg once-daily doses for 8–12 weeks, followed by nephrectomy. Adult patients with localized ccRCC, radiological evidence of nonmetastatic disease, and were appropriate candidates for nephrectomy were eligible. A complete set of eligibility criteria is provided in the Supplemental Data. Patients were required to have a performance status of 0 or 1 with adequate organ function and no known coagulopathy. Patients who had a known or suspected allergy to pazopanib, were unable to swallow oral medication, were pregnant or breastfeeding, or had a history of a cerebrovascular accident or cardiovascular condition within 6 months of starting pazopanib were excluded. Other eligibility criteria are included in the Supplemental Data.

Imaging was performed on all patients before therapy and before nephrectomy; the primary endpoint of tumor response was measured using RECIST 1.1 (30).

### Laboratory correlatives

#### Tissue and blood samples.

In consenting patients, tissue biopsies were performed before starting treatment and from nephrectomy specimens. Tissue cores were flash frozen and stored in liquid nitrogen. Whole blood was also collected for genomic studies.

#### Genomic analysis and transcriptional analysis.

Samples from the pretreatment and postnephrectomy biopsies were divided for immediate snap freezing or formalin fixation, followed by processing using a standard tissue processor (Leica Peloris II) and paraffin embedding. Five micron-thick sections were stained with H&E for light microscopic evaluation. Images from these diagnostic slides were prepared at 40× magnification on a Leica digital imaging microscope using standard bright-field settings.

Samples were processed for DNA from pulverized frozen tissue or whole blood buffy coat using QIAGEN genomic DNA isolation products. DNA was fragmented by sonication, and libraries were prepared using Nextera Rapid Exome Capture (Illumina). The samples were run on Illumina 2500 HiSeq, using 100 bp paired end reads. Alignment to hg19 was performed using the BWA “mem” algorithm (available at https://github.com/lh3/bwa). Primary alignments were then realigned using ABRA (31); somatic variants were called using Strelka (32) and were annotated with SnpEff (33). False negatives could greatly influence our inference regarding the frequency and nature of private mutations. Thus, we chose to limit false negatives by first selecting all mutations called with high confidence (Strelka QSS_NT>30 or QSI_NT>30) in any 1 sample from a subject. The union set from all samples of a subject was then used to query the data and estimate the mutant allele frequency for all samples of a subject. In this way, any evidence (reads) supporting a mutation in both pre- and posttreatment samples was used even if a high-quality mutation call exits in only one of the pair. The resulting matrix of mutant allele frequencies were characterized with hierarchical clustering (Euclidean distance, complete linkage), and distributions were compared visually with quantile-quantile plots. Private mutations are defined here as mutations that are uniquely observed in a single tumor sample and not shared by any other tumor sample from the same subject. A Kolmogorov-Smirnov test was performed to test the null hypothesis that the distribution of mutant allele frequencies was not different between the pre- and posttreatment samples. Differences could be due to change in tumor cellularity or clonality. In order to control for differences in tumor cellularity, the same test was performed using only mutations with frequency greater than zero in both samples.

The whole exome data were used to calculate allele frequencies using the DNAcopy package in R and present as copy number for comparison between pre- and posttreatment. Although pretreatment biopsy was a requirement for enrollment, only 5 of the subjects had pretreatment biopsies of sufficient quantity and quality for nucleic acid assessment.

#### Transcriptional analysis.

Samples were processed for RNA from pulverized frozen tissue using QIAGEN RNA isolation products. RNA was quantified using Qubit, and quality was assured using NanoDrop to assess potential contamination and TapeStation to assess fragment size. RNA sequencing libraries were prepared using the Illumina TruSeq stranded protocol, and sequencing was done using the Illumina HiSeq2500 platform with 2 × 75 paired end chemistry. Output Fastq files were assessed for sequencing read quality using the FASTQC software (34), and Fastq files were aligned to the hg38 transcriptome with STAR (v2.4.2a) (35) and assembled with Salmon (v0.6.0) (36). Samples with fewer than 30 million multimapped and uniquely mapped reads were dropped from further analysis. Genes were converted from UCSC format to HGNC symbols and Entrez IDs using the R package biomRt (37). Differential gene expression was done using the R package DESeq2 (v1.14.1) (38) on the unnormalized gene output. GSEA was performed on upper quartile normalized gene counts using the single-sample GSEA (ssGSEA) method in the R package GSVA (v1.22.4) (39). Most gene sets came from Molecular Signatures Database’s (MSigDB’s) chemical and genetic perturbations (https://www.gsea-msigdb.org/gsea/msigdb/), KEGG (https://www.genome.jp/kegg/pathway.html), oncogenetic signatures (40), and previously curated immune gene signature gene sets (41–50). All next-generation sequencing data have been deposited in a MINSEQE-compliant public database (dbGap; accession hon. phs002053.v1.p1; https://www.ncbi.nlm.nih.gov/projects/gap/cgi-bin/study.cgi?study_id=phs002053.v1.p1).

### Statistics

The overall RR was calculated and reported, along with its exact 95% CI. The Kaplan-Meier method was used to estimate RFS and OS, and their estimates at 1 year — along with their 95% CIs — have been reported. RFS has been calculated using the time from the start date of treatment until the date of documented disease recurrence (as defined via RECIST1.1), to the date of death from any cause, or to the date of last contact (censored). OS has been calculated using the time from the start date of treatment to the date of death from any cause or the date of last contact (censored). The Wilcoxon rank sum test was used for 2-group comparisons. The Kolmogorov-Smirnov test was used to compare distributions. The 2-tailed, paired *t* test was used to make within-patient pre- to posttreatment comparisons. The Benjamini-Hochberg FDR method was used to adjust for multiple testing. Toxicity assessments were graded according to the NCI Common Toxicity Criteria, version 4.0 (CTCAE v4), and were reported as percentages of the highest grade toxicity, per toxicity, per patient. Reported *P* values were 2 sided, with *P* < 0.05 considered statistically significant. Statistical analyses were performed with SAS statistical software, version 9.2, and R: A language and environment for statistical computing (R Foundation for Statistical Computing; ISBN 3-900051-07-0; http://www.R-project.org/). The Morisita-Horn index was used to compare the similarity of adaptive immune receptor repertoires between samples. This similarity index incorporates the number of shared clonotypes, as well as the relative dominance of shared clonotypes, and it is thus well suited to adaptive immune receptor repertoire data. The Morisita-Horn index values were calculated using the “horn” method of the R function vegan::vegdist (https://CRAN.R-project.org/package=vegan).

### Study approval

This clinical trial was approved by the IRBs at the University of Carolina at Chapel Hill (Office of Human Research Ethics) and MD Anderson (Office of Human Subjects Protection), and the research was conducted according to the Declaration of Helsinki principles. All participants provided written informed consent before the initiation of any research procedures.

## Author contributions

CGW contributed by designing research studies, conducting experiments, acquiring data, analyzing data, and writing the manuscript. JEF contributed by conducting experiments, acquiring data, and writing the manuscript. JSP contributed by designing research studies, analyzing data, and writing the manuscript. DTM contributed by designing research studies, analyzing data, and writing the manuscript. JGW contributed by writing the manuscript. SJM contributed by acquiring data, providing reagents, and writing the manuscript. EMW contributed by designing research studies, conducting experiments, acquiring data, and writing the manuscript. WYK contributed by designing research studies, conducting experiments, acquiring data, and writing the manuscript. MIM contributed by designing research studies and writing the manuscript. KEB contributed by designing research studies, analyzing data, and writing the manuscript. NBD contributed by designing research studies, analyzing data, and writing the manuscript. SMH contributed by designing research studies, analyzing data, and writing the manuscript. JAK contributed by designing research studies, conducting experiments, acquiring data, analyzing data, and writing the manuscript. DSB contributed by conducting experiments, acquiring data, analyzing data, and writing the manuscript. BGV contributed by designing research studies, conducting experiments, acquiring data, analyzing data, providing reagents, and writing the manuscript. TP contributed by designing research studies, analyzing data, providing reagents, and writing the manuscript. WKR contributed by designing research studies, conducting experiments, acquiring data, analyzing data, providing reagents, and writing the manuscript

## Supplementary Material

supplemental data

trial reporting checklists

ICMJE disclosure forms

## Figures and Tables

**Table 1:**
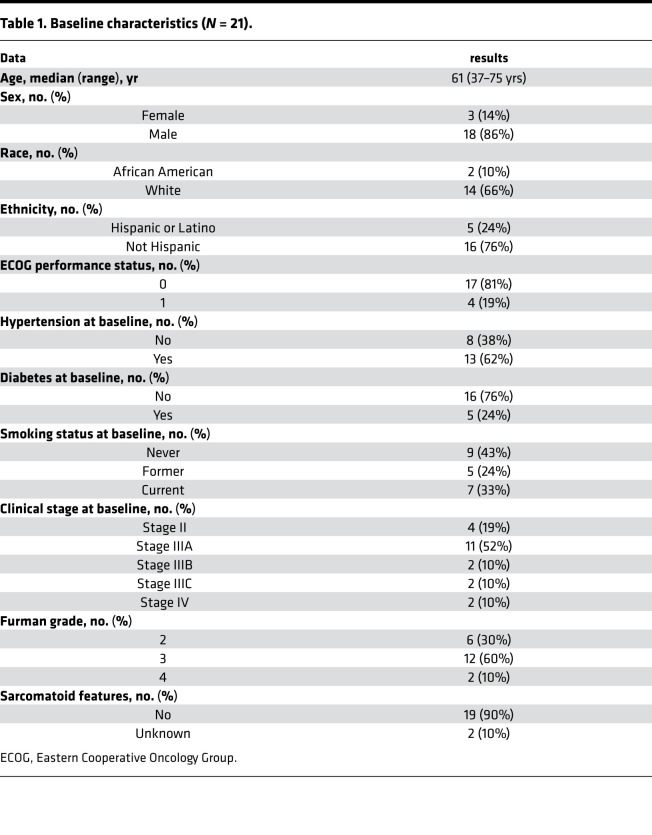
Baseline characteristics (*N* = 21).

**Table 2:**
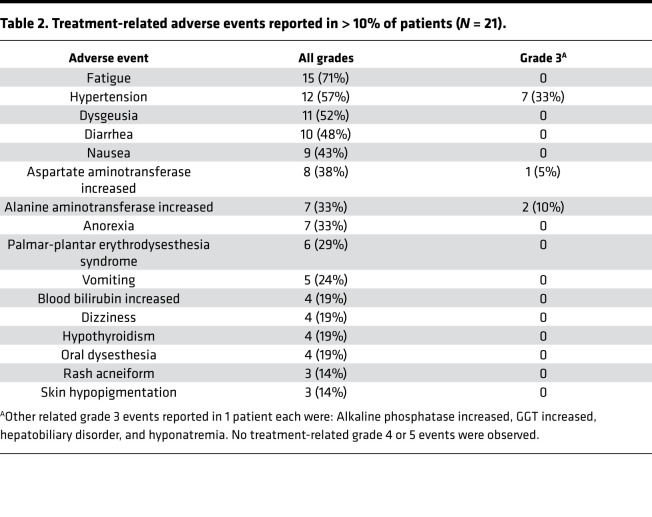
Treatment-related adverse events reported in > 10% of patients (*N* = 21).

## Version 1 11/19/2020

Electronic publication
