# Supplementary material for: Neoadjuvant pazopanib and molecular analysis of tissue response in renal cell carcinoma
Source: JCI Insight. 2020 Nov 19;5(22):e132852. doi: 10.1172/jci.insight.132852 (PMC7710285; doi:10.1172/jci.insight.132852)

SUPPLEMENT

Complete Eligibility Criteria

Inclusion Criteria

Subject must meet all of the following inclusion criteria to participate in this study:

- Age ≥ 18 years with radiographic evidence of nonmetastatic renal cell carcinoma
- Histological verification of clear cell renal cell carcinoma (Note: this will be confirmed post informed consent, see schema)
- Clinical stage 2 or greater with localized disease
- No evidence of extranodal metastatic disease
- Appropriate candidate for surgery
- ECOG Performance status of 0-1
- Adequate organ function as evidenced by:
  - Absolute neutrophil count ≥1.5 x 10^9/L
  - Hemoglobin ≥9 g/dL (without transfusion within 7 days of assessment)
  - Platelets ≥100x 10^9/L
  - Serum creatinine ≤1.5x ULN or calculated creatinine clearance ≥50mL/min
  - Hepatic Assessment
    - Total Bilirubin ≤1.5x ULN
    - AST ≤1.5x ULN
    - ALT ≤1.5x ULN
    - Concomitant elevations in bilirubin and AST/ALT above 1.0 x ULN not permitted.
  - PT or INR ≤1.2 x ULN
  - aPTT ≤ 1.2 x ULN
  - Urine Protein to Creatinine Ratio (UPC) <1 (NOTE, if ≥1, then a 24-hour urine protein must be assessed: if 24-hour urine protein >1g, patient ineligible
- Serum calcium, magnesium, potassium within normal limits, or if outside of normal limits, must be deemed clinically insignificant by the Investigator.
- No known coagulopathy
- Ability to read and follow instructions
- Women of childbearing potential must have a negative serum pregnancy test performed within 14 days prior to the start of pazopanib treatment and both men and women must be willing to use adequate contraception.

- Able to provide written, informed consent
- Blood and urine samples must be provided from all subjects for biomarker analysis before and during treatment with pazopanib

Exclusion Criteria

Subjects meeting any of the exclusion criteria listed below at baseline will be excluded from study participation: these drugs will be excluded from the protocol.

- Known or suspected allergy to pazopanib
- Inability to swallow or retain oral medication
- **Prior malignancy**. Exception: Subjects who have had another malignancy and have been disease-free for three years, or subjects with a history of completely resected non-melanomatous skin carcinoma or successfully treated in situ carcinoma are eligible.
- Unable or unwilling to discontinue use of prohibited medications for at least 7 days prior to the first dose of study drug and for the duration of the study.
- Clinically significant gastrointestinal abnormalities that may increase the risk for gastrointestinal bleeding including, but not limited to:
    - Active peptic ulcer disease
    - Inflammatory bowel disease (e.g. ulcerative colitis, Chrohn’s disease), or other gastrointestinal conditions with increased risk of perforation
    - History of abdominal fistula, gastrointestinal perforation, or intra-abdominal abscess within 28 days prior to beginning study treatment
    - Clinically significant hemoptysis or gastrointestinal hemorrhage in the past 6 months
- Clinically significant gastrointestinal abnormalities that may affect absorption of investigational product including, but not limited to:
    - Malabsorption syndrome
    - Major resection of the stomach or small bowel.
- History of cerebrovascular accident including transient ischemic attack (TIA), pulmonary embolism or untreated deep venous thrombosis (DVT) within the past 6 months; Note: Subjects with recent DVT who have been treated with therapeutic anti-coagulating agents for at least 6 weeks are eligible but must be monitored regularly for changes in relevant coagulation parameters as clinically indicated as well as any clinical bleeding episodes.

- History of any one or more of the following cardiovascular conditions within the past 6 months:
    - Cardiac angioplasty or stenting
    - Myocardial infarction
    - Unstable angina
    - Coronary artery bypass graft surgery
    - Symptomatic peripheral vascular disease
    - Class III or IV congestive heart failure, as defined by the New York Heart Association (NYHA)
- Hypertension [defined as systolic blood pressure (SBP) of ≥140 mmHg OR diastolic blood pressure (DBP) of ≥ 90mmHg] in spite of optimal medical management. Note: Initiation or adjustment of antihypertensive medication(s) is permitted prior to study entry. In this event, BP must be re-assessed on two occasions that are separated by a minimum of 1 hour; on each of these occasions, the mean (of 3 readings) SBP / DBP values from each BP assessment must be <140/90 mmHg in order for a subject to be eligible for the study.
- Evidence of active bleeding or bleeding diathesis
- Any serious and/or unstable pre-existing medical (especially hepatic disease), psychiatric, or other condition that could interfere with subject’s safety, provision of informed consent, or compliance to study procedures.
- Prior major surgery or trauma within 28 days prior to first dose of pazopanib and/or presence of any non-healing wound, fracture, or ulcer (procedures such as catheter placement not considered to be major).
- Pregnant or breastfeeding; breastfeeding may not resume for 14 days after the last dose of pazopanib
- Prior treatment with any of the following anti-cancer therapies for treatment of their RCC:
    - radiation therapy, surgery or tumor embolization
    - chemotherapy, immunotherapy, biologic therapy, investigational therapy or hormonal therapy
- Administration of any non-oncologic investigational drug within 30 days or 5 half-lives whichever is longer prior to receiving the first dose of study treatment.
- Baseline QTc>480 msec or other clinically significant baseline ECG abnormality

Supp. Fig. S1. Copy number analysis. Copy number variation was calculated from the whole exome sequencing data. The copy number profile is displayed for each case, labeled on the left. Black indicates the pre-treatment biopsy, red indicates the post-treatment sample. Comparing the variation patterns over the genome, the profiles were highly similar between paired samples.

Supp. Figure S2. Scatter plots of mutant allele frequencies for pre- and post-treatment samples. One scatter plot for each case is shown, and labeled with case number in the upper left corner. Each data point represents a detected mutation allele frequency in either the pre-treatment sample (x-axis) or the post-treatment sample (y-axis). Black circles: non-synonymous mutations. Grey circles: synonymous mutations. Genes identified by the TCGA as high frequency for clear cell renal cell carcinoma are labeled.

Supp. Fig. S3. Focused transcript analysis. RNA seq data from pre- and post-treatment samples was analyzed for the expression of annotated gene sets known to be associated with hypoxia or hypoxia inducible factor expression, a common feature of VHL-deficient clear cell renal cell carcinoma. The majority of expression signatures showed no significant difference between pre- and post-treatment samples. Error bars represent standard deviation. $^{**}p<0.005$.

Supplement References:

1. Mose, L. E., Wilkerson M. D., Hayes D. N., et al. ABRA: improved coding indel detection via assembly-based realignment. Bioinformatics 2014;30:2813.
2. Saunders, C. T., Wong W. S., Swamy S., et al. Strelka: accurate somatic small-variant calling from sequenced tumor-normal sample pairs. Bioinformatics 2012;28:1811.
3. Cingolani, P., Platts A., Wang le L., et al. A program for annotating and predicting the effects of single nucleotide polymorphisms, SnpEff: SNPs in the genome of Drosophila melanogaster strain w1118; iso-2; iso-3. Fly (Austin) 2012;6:80.
4. Dobin, A., Davis C. A., Schlesinger F., et al. STAR: ultrafast universal RNA-seq aligner. Bioinformatics 2013;29:15.
5. Patro, R., Duggal G., Love M. I., et al. Salmon provides fast and bias-aware quantification of transcript expression. Nat Methods 2017;14:417.
6. Durinck, S., Spellman P. T., Birney E., et al. Mapping identifiers for the integration of genomic datasets with the R/Bioconductor package biomaRt. Nat Protoc 2009;4:1184.

Supp. Fig. S1. Copy number analysis. Copy number variation was calculated from the whole exome sequencing data. The copy number profile is displayed for each case, labeled on the left. Black indicates the pre-treatment biopsy, red indicates the post-treatment sample. Comparing the variation patterns over the genome, the profiles were highly similar between paired samples.

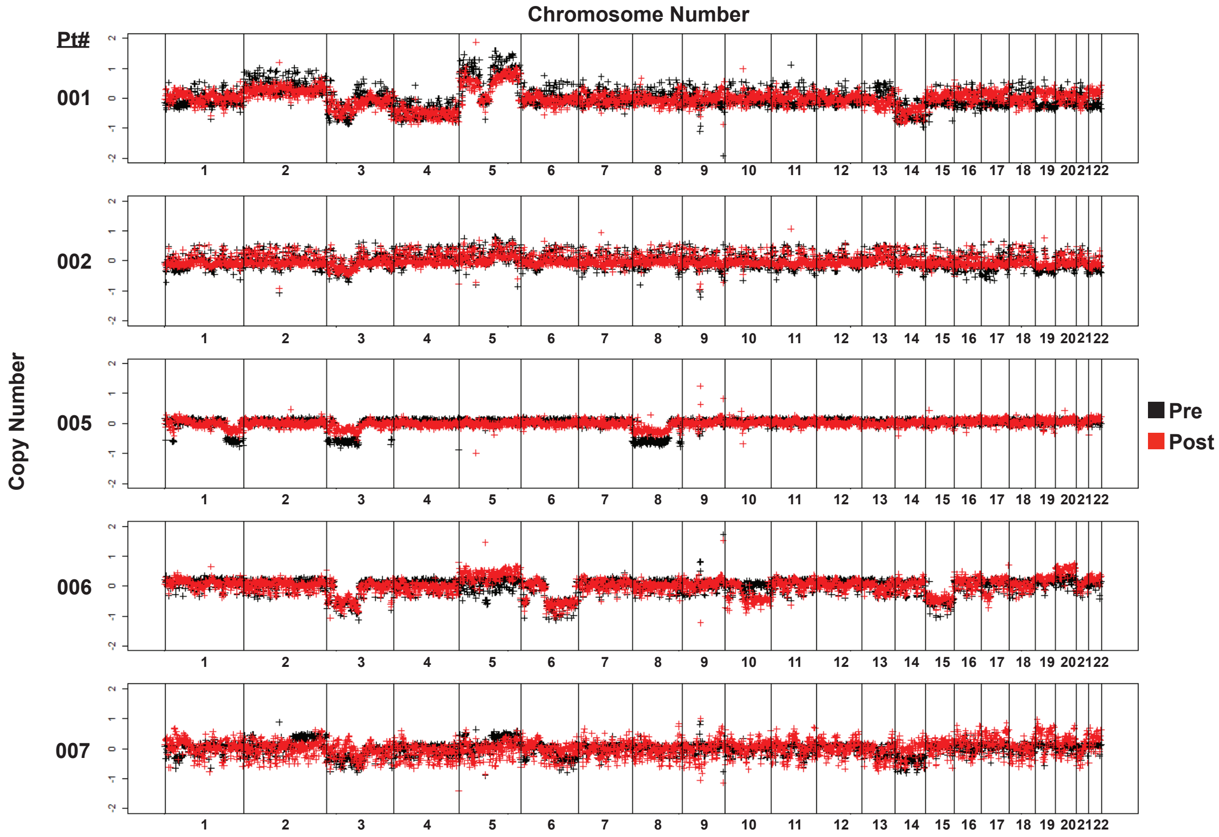

Supp. Figure S2. Scatter plots of mutant allele frequencies for pre- and post-treatment samples. One scatter plot for each case is shown, and labeled with case number in the upper left corner. Each data point represents a detected mutation allele frequency in either the pre-treatment sample (x-axis) or the post-treatment sample (y-axis). Black circles: non-synonymous mutations. Grey circles: synonymous mutations. Genes identified by the TCGA as high frequency for clear cell renal cell carcinoma are labeled.

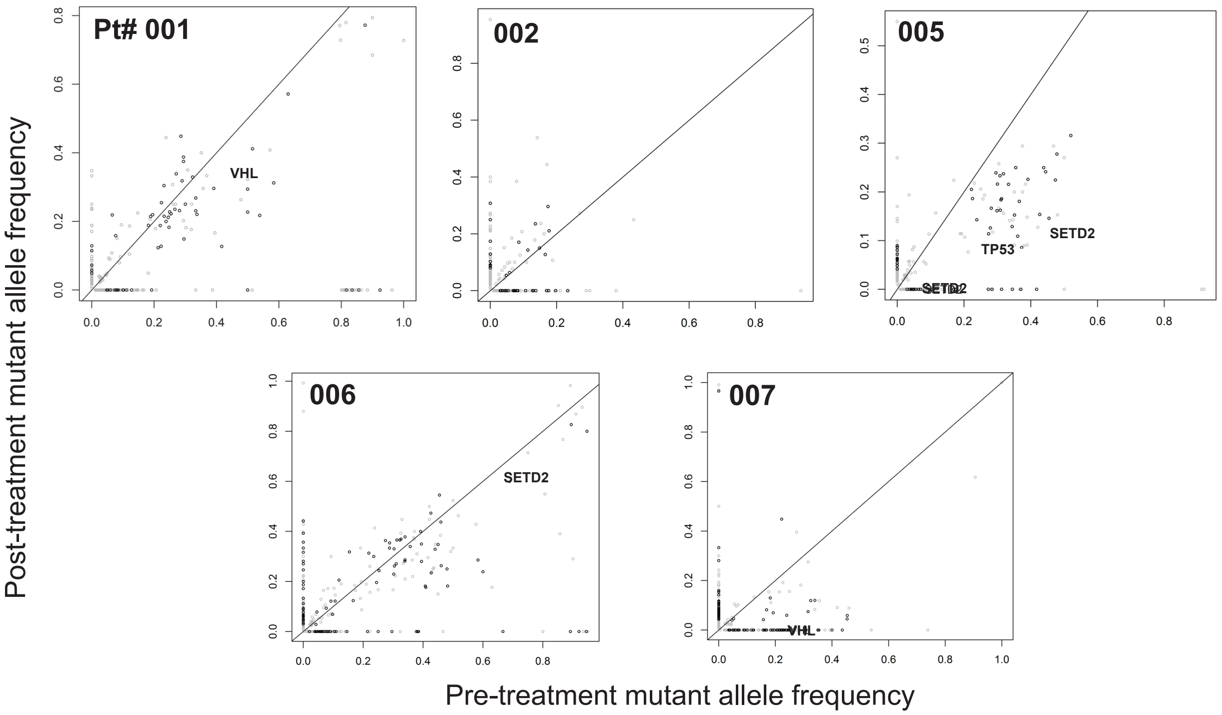

Supp. Fig. S3. Focused transcript analysis. RNA seq data from pre- and post-treatment samples was analyzed for the expression of annotated gene sets known to be associated with hypoxia or hypoxia inducible factor expression, a common feature of VHL-deficient clear cell renal cell carcinoma. The majority of expression signatures showed no significant difference between pre- and post-treatment samples. Error bars represent standard deviation. **p<0.005.

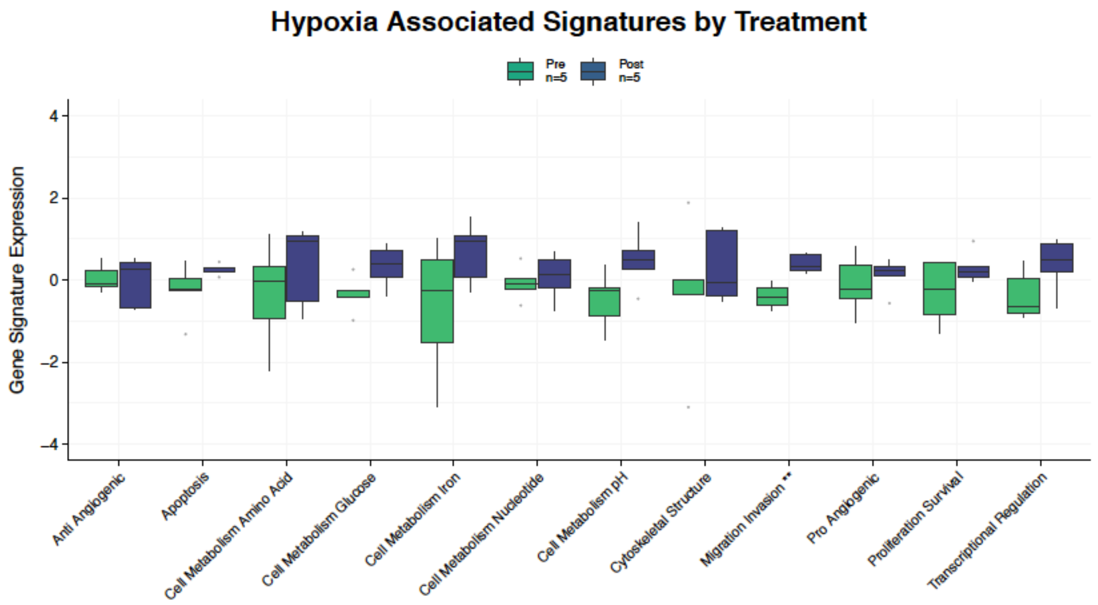

Supplement: supplemental data [file jciinsight-5-132852-s047.pdf]
